# Correction: Identifying suitable tester for evaluating Striga resistant lines using DArTseq markers and agronomic traits

**DOI:** 10.1371/journal.pone.0317847

**Published:** 2025-01-16

**Authors:** Degife Zebire, Abebe Menkir, Victor Adetimirin, Wende Mengesha, Silvestro Meseka, Melaku Gedil

There is an error in the article title. The correct title is: Identifying Suitable Tester for Evaluating Striga Resistant Maize Inbred Lines using DArTseq Markers and Agronomic Traits. The correct citation is: Zebire D, Menkir A, Adetimirin V, Mengesha W, Meseka S, Gedil M (2021) Identifying Suitable Tester for Evaluating Striga Resistant Maize Inbred Lines using DArTseq Markers and Agronomic Traits. PLoS ONE 16(6): e0253481. https://doi.org/10.1371/journal.pone.0253481.
