# Supplementary material for: Identifying suitable tester for evaluating Striga resistant lines using DArTseq markers and agronomic traits
Source: PLoS One. 2021 Jun 18;16(6):e0253481. doi: 10.1371/journal.pone.0253481 (PMC8213128; doi:10.1371/journal.pone.0253481)
Supplement: S1 Table — (DOCX) [file pone.0253481.s001.docx]

S1 Table:

L1-L30 = *Striga* resistant inbred lines and T1-T3 = Testers with varying levels of *Striga* resistance reaction described in Supplementary Table 1
