# Supplementary material for: Identifying suitable tester for evaluating Striga resistant lines using DArTseq markers and agronomic traits
Source: PLoS One. 2021 Jun 18;16(6):e0253481. doi: 10.1371/journal.pone.0253481 (PMC8213128; doi:10.1371/journal.pone.0253481)
Supplement: S2 Table — (DOCX) [file pone.0253481.s002.docx]

S2 Table:

|  | Testers | | | | | |
| --- | --- | --- | --- | --- | --- | --- |
| Inbred lines | TZISTR1207(T1) | | TZSTRI106 (T2) | | TZISTR1033 (T3) | |
|  | GD | PD | GD | PD | GD | PD |
| TZISTR1224 (L1) | 0.34 | 0.10 | 0.33 | 0.13 | 0.39 | 0.63 |
| TZISTR1215 (L2) | 0.33 | 0.21 | 0.31 | 0.11 | 0.38 | 0.34 |
| TZISTR1220 (L3) | 0.37 | 0.18 | 0.02 | 0.12 | 0.41 | 0.63 |
| TZISTR1222 (L4) | 0.36 | 0.32 | 0.29 | 0.20 | 0.41 | 0.15 |
| TZISTR1223 (L5) | 0.36 | 0.49 | 0.32 | 0.21 | 0.39 | 0.44 |
| TZISTR1225 (L6) | 0.33 | 0.09 | 0.34 | 0.24 | 0.40 | 0.68 |
| TZISTR1226 (L7) | 0.34 | 0.30 | 0.31 | 0.17 | 0.39 | 0.30 |
| TZISTR1227 (L8) | 0.44 | 0.49 | 0.45 | 0.56 | 0.39 | 0.28 |
| TZISTR1228 (L9) | 0.39 | 0.17 | 0.41 | 0.22 | 0.39 | 0.50 |
| TZISTR1230 (L10) | 0.35 | 0.11 | 0.32 | 0.22 | 0.39 | 0.40 |
| TZISTR1231 (L11) | 0.32 | 0.09 | 0.33 | 0.23 | 0.39 | 0.26 |
| TZISTR1232 (L12) | 0.33 | 0.28 | 0.31 | 0.25 | 0.39 | 0.82 |
| TZISTR1235 (L13) | 0.33 | 0.49 | 0.26 | 0.36 | 0.39 | 0.28 |
| TZISTR1236 (L14) | 0.33 | 0.05 | 0.31 | 0.23 | 0.39 | 0.55 |
| TZISTR1237 (L15) | 0.35 | 0.29 | 0.30 | 0.30 | 0.39 | 0.06 |
| TZISTR1238 (L16) | 0.36 | 0.18 | 0.32 | 0.06 | 0.40 | 0.38 |
| TZISTR1211 (L17) | 0.34 | 0.30 | 0.31 | 0.33 | 0.38 | 0.24 |
| TZISTR1214 (L18) | 0.36 | 0.18 | 0.33 | 0.17 | 0.40 | 0.35 |
| TZISTR1216 (L19) | 0.35 | 0.18 | 0.32 | 0.28 | 0.38 | 0.32 |
| TZISTR1217 (L20) | 0.36 | 0.27 | 0.31 | 0.35 | 0.38 | 0.20 |
| TZISTR1218 (L21) | 0.36 | 0.08 | 0.28 | 0.22 | 0.39 | 0.27 |
| TZISTR1233 (L22) | 0.37 | 0.42 | 0.40 | 0.54 | 0.38 | 0.13 |
| TZSTRI109 (L23) | 0.43 | 0.24 | 0.45 | 0.40 | 0.39 | 0.38 |
| TZSTRI110 (L24) | 0.34 | 0.30 | 0.22 | 0.12 | 0.37 | 0.37 |
| TZSTRI112 (L25) | 0.43 | 0.46 | 0.45 | 0.57 | 0.39 | 0.34 |
| TZSTRI113 (L26) | 0.40 | 0.20 | 0.42 | 0.41 | 0.37 | 0.38 |
| TZSTRI114 (L27) | 0.42 | 0.40 | 0.42 | 0.44 | 0.39 | 0.19 |
| TZISTR1028 (L28) | 0.38 | 0.22 | 0.41 | 0.22 | 0.34 | 0.42 |
| TZISTR1029 (L29) | 0.38 | 0.60 | 0.38 | 0.39 | 0.34 | 0.80 |
| TZISTR1030 (L30) | 0.36 | 0.58 | 0.39 | 0.39 | 0.34 | 0.33 |
| **min** | 0.32 | 0.05 | 0.02 | 0.06 | 0.34 | 0.06 |
| **max** | 0.44 | 0.60 | 0.45 | 0.57 | 0.41 | 0.82 |
| **Mean** | 0.36 | 0.27 | 0.33 | 0.28 | 0.38 | 0.38 |
