# Supplementary material for: Identifying suitable tester for evaluating Striga resistant lines using DArTseq markers and agronomic traits
Source: PLoS One. 2021 Jun 18;16(6):e0253481. doi: 10.1371/journal.pone.0253481 (PMC8213128; doi:10.1371/journal.pone.0253481)
Supplement: S3 Table — (DOCX) [file pone.0253481.s003.docx]

S3 Table:

|  | *Striga* infested | | | | | | | | | | |
| --- | --- | --- | --- | --- | --- | --- | --- | --- | --- | --- | --- |
| Traits | ASI | DYSK | DYAN | EASP | EPP | PLHT | STRCO1 | STRCO2 | STRRAT1 | STRRAT2 | YLD |
| ASI |  | 0.50^†^ | 0.39^***^ | 0.20 | -0.12 | 0.13 | -0.13 | -0.02 | 0.01 | 0.11 | -0.06 |
| DYSK |  |  | 0.99^†^ | 0.21^*^ | -0.10 | 0.36^***^ | -0.37^***^ | -0.23^*^ | -0.23^*^ | -0.18 | 0.15 |
| DYAN |  |  |  | 0.20^*^ | -0.12 | 0.36^***^ | -0.35^***^ | -0.23^*^ | -0.23^*^ | -0.19 | 0.15 |
| EASP |  |  |  |  | -0.71^†^ | -0.22^*^ | 0.25^**^ | 0.25^**^ | 0.60^†^ | 0.62^†^ | -0.76^†^ |
| EPP |  |  |  |  |  | 0.23 | -0.47^†^ | -0.45^†^ | -0.74^†^ | -0.71^†^ | 0.72^†^ |
| PL HT |  |  |  |  |  |  | -0.4^†^ | -0.36^***^ | -0.38^***^ | -0.31 | 0.50^†^ |
| STRCO1 |  |  |  |  |  |  |  | 0.89^†^ | 0.54^†^ | 0.55^†^ | -0.53^†^ |
| STRCO2 |  |  |  |  |  |  |  |  | 0.46^†^ | 0.54^†^ | -0.47^†^ |
| STRRAT1 |  |  |  |  |  |  |  |  |  | 0.88^†^ | -0.68^†^ |
| STRRAT2 |  |  |  |  |  |  |  |  |  |  | -0.69^†^ |
| YLD |  |  |  |  |  |  |  |  |  |  |  |
|  | *Striga* non-infested | | | | | | | | | | |
|  | ASI | DYSK | DYAN | EASP | EHT | EPP | HUSK | PASP | PLHT | YLD |  |
| ASI |  | 0.42^†^ | 0.28^**^ | 0.22^*^ | 0.04 | -0.03 | 0.23^*^ | 0.19 | 0.00 | -0.12 |  |
| DYSK |  |  | 0.99^†^ | 0.25^**^ | 0.28^**^ | -0.31^**^ | -0.04 | 0.26^**^ | 0.30^**^ | 0.10 |  |
| DYAN |  |  |  | 0.23^*^ | 0.29^**^ | -0.31^**^ | -0.08 | 0.24^*^ | 0.32^**^ | 0.13 |  |
| EASP |  |  |  |  | -0.08 | -0.23^*^ | 0.27^**^ | 0.25^*^ | -0.12 | -0.47^†^ |  |
| EHT |  |  |  |  |  | -0.04 | -0.23^*^ | -0.11 | 0.80^†^ | 0.43^†^ |  |
| EPP |  |  |  |  |  |  | -0.15 | -0.29^**^ | 0.04 | 0.18 |  |
| HUSK |  |  |  |  |  |  |  | 0.36^***^ | -0.28^**^ | -0.34^***^ |  |
| PASP |  |  |  |  |  |  |  |  | -0.07 | -0.22^*^ |  |
| PL HT |  |  |  |  |  |  |  |  |  | 0.54^†^ |  |
| YLD |  |  |  |  |  |  |  |  |  |  |  |

*, **, ***, † Significant at p < 0.05, 0.01, 0.001 and 0.0001 levels, respectively. DYSK= Days to 50% silking; DYAN= Days to 50% anthesis; PLHT= Plant height (cm); STRRAT1 and STRRAT2= *Striga* damage rating at 8 and 10 WAP, respectively; STRCO1 and STRCO2= *Striga* emergence count at 8 and 10 WAP, respectively; EASP = ear aspect; EHT = ear height; PASP = plant aspect; HUSK = husk cover; ASI = Anthesis silking interval; EPP = ears per plant and YLD = grain yield (kg/ha).
