# Supplementary material for: Identifying suitable tester for evaluating Striga resistant lines using DArTseq markers and agronomic traits
Source: PLoS One. 2021 Jun 18;16(6):e0253481. doi: 10.1371/journal.pone.0253481 (PMC8213128; doi:10.1371/journal.pone.0253481)
Supplement: S4 Table — (DOCX) [file pone.0253481.s004.docx]

S4 Table:

| Crosses | DYSK | DYAN | PL HT | RAT1 | RAT2 | CO1 | CO2 | EASP | EPP | YLD |
| --- | --- | --- | --- | --- | --- | --- | --- | --- | --- | --- |
| L1×T1 | 0.64 | 0.73 | -2.84 | -0.19 | -0.08 | -8.71 | -7.7 | -0.05 | 0.02 | -145.3 |
| L2×T1 | 0.18 | 0.27 | 2.45 | 0.19 | -0.41 | -3.38 | -7.24 | 0.03 | -0.04 | -343.9 |
| L3×T1 | -0.61 | -0.48 | -2.14 | 0.14 | 0.3 | -0.09 | -9.07 | -0.22 | 0.02 | 295.7 |
| L4×T1 | 0.60 | 0.43 | -4.01 | -0.31 | -0.49 | 10.83 | 14.76 | 0.07 | 0.05 | -303.1 |
| L5×T1 | 0.14 | 0.18 | -2.72 | 0.48^*^ | 0.55 | 5.29 | 7.55 | 0.03 | -0.02 | -142.3 |
| L6×T1 | 0.68 | 0.77 | 5.28 | 0.14 | 0.01 | 8.46 | 6.26 | 0.15 | 0.02 | -305.4 |
| L7×T1 | -0.07 | -0.11 | 2.91 | -0.40 | -0.37 | -9.34 | -18.99 | -0.14 | 0.00 | 125.1 |
| L8×T1 | -0.63 | -0.98 | 5.03 | 0.02 | -0.12 | -8.13 | 2.76 | -0.18 | 0.12 | 438.8 |
| L9×T1 | -0.19 | -0.15 | 0.28 | 0.02 | -0.53 | -1.88 | -1.95 | -0.16 | 0.02 | 478.5 |
| L10xT1 | 0.18 | 0.35 | 5.49 | 0.19 | 0.13 | -9.42 | -5.45 | -0.14 | -0.05 | 348.9 |
| L11×T1 | 0.01 | 0.18 | 0.57 | -0.44 | -0.45 | -7.71 | -7.11 | -0.03 | -0.00 | -63.8 |
| L12×T1 | 0.93 | 0.68 | -2.80 | -0.02 | -0.49 | -3.42 | -5.16 | 0.11 | 0.01 | -221.8 |
| L13×T1 | 0.68 | 0.60 | -3.93 | -0.11 | 0.22 | 4.37 | 12.39 | 0.16 | -0.00 | -190 |
| L14×T1 | -0.15 | -0.11 | -1.59 | -0.15 | 0.30 | 12.62^*^ | 26.22^*^ | 0.03 | -0.02 | 20.1 |
| L15×T1 | 0.18 | 0.23 | -6.47 | 0.06 | -0.16 | 2.79 | -1.49 | 0.26^*^ | 0.04 | -309 |
| L16×T1 | 0.60 | 0.77 | -2.47 | 0.39 | 0.47 | 1.00 | -3.41 | 0.30^**^ | 0.02 | -504.3 |
| L17×T1 | 0.60 | 0.64 | -5.34 | -0.44 | 0.01 | -4.21 | -1.61 | -0.18 | 0.02 | 165.3 |
| L18×T1 | 0.18 | 0.02 | 2.86 | -0.02 | -0.12 | -2.46 | -16.28 | 0.18 | 0.03 | 45.9 |
| L19×T1 | -0.82 | -0.44 | -1.14 | 0.02 | -0.12 | 10.54 | 9.18 | -0.05 | -0.05 | 120.5 |
| L20×T1 | -0.24 | -0.32 | -6.05 | 0.81^***^ | 1.09^**^ | 20.87^**^ | 20.39 | 0.07 | -0.03 | -721.8^**^ |
| L21×T1 | -1.03^*^ | -0.98^*^ | 4.41 | -0.15 | -0.16 | 4.66 | 6.39 | -0.05 | 0.01 | 152.6 |
| L22×T1 | 0.43 | 0.31 | -4.01 | 0.02 | -0.08 | -5.34 | -14.11 | 0.01 | -0.04 | -503.9 |
| L23×T1 | 0.51 | 0.43 | 0.95 | -0.06 | -0.20 | -13.7^*^ | -19.11 | 0.20 | 0.03 | -384.4 |
| L24×T1 | -0.82 | -0.86 | 1.16 | -0.19 | 0.05 | 1.08 | 6.59 | 0.09 | 0.05 | -409.6 |
| L25×T1 | 0.18 | 0.02 | -2.47 | 0.56^*^ | 0.80^*^ | 6.33 | 7.80 | 0.18 | -0.08 | -564.4^*^ |
| L26×T1 | 0.39 | 0.10 | -1.55 | 0.06 | 0.34 | -8.96 | -4.11 | -0.14 | 0.01 | 363.1 |
| L27×T1 | 0.26 | 0.18 | 2.82 | -0.11 | -0.24 | 5.75 | 6.76 | -0.05 | 0.03 | 192.1 |
| L28×T1 | -1.19^*^ | -0.90^**^ | 3.91 | 0.31 | 0.17 | -1.46 | -3.86 | -0.07 | 0.06 | 488.1 |
| L29×T1 | -0.24 | -0.57 | 5.28 | -0.44 | 0.01 | -0.50 | 7.18 | -0.30^**^ | -0.18^*^ | **1085.6^***^** |
| L30×T1 | -1.24^*^ | -0.98^*^ | 6.16 | -0.40 | -0.41 | -5.92 | -7.57 | -0.14 | 0.00 | **792.6^**^** |
| L1×T2 | -0.42 | -0.32 | 0.29 | -0.06 | -0.34 | 3.95 | 6.41 | 0.05 | -0.04 | 44 |
| L2×T2 | -0.38 | -0.40 | -2.92 | -0.31 | -0.05 | 6.28 | 8.24 | 0.20 | 0.01 | -56.7 |
| L3×T2 | 0.83 | 0.85 | 1.37 | 0.28 | 0.28 | 6.20 | 13.78 | 0.45^***^ | -0.05 | -829.6^**^ |
| L4×T2 | -0.47 | -0.36 | 2.12 | 0.44 | 0.62 | -12.6^*^ | -13.38 | -0.14 | -0.05 | 280.1 |
| L5×T2 | -0.30 | 0.02 | 6.54 | -0.26 | -0.09 | -6.18 | -13.59 | -0.05 | 0.02 | 110.5 |
| L6×T2 | -0.88 | -0.90^***^ | 4.41 | -0.22 | -0.51 | -5.39 | -7.88 | -0.18 | 0.00 | **830.1^**^** |
| L7×T2 | 0.49 | 0.35 | 2.66 | -0.14 | 0.12 | 7.57 | 5.24 | 0.09 | -0.01 | 170 |
| L8×T2 | 0.68 | -0.15 | 1.66 | -0.35 | -0.26 | -9.09 | -13.88 | -0.20 | 0.03 | 195.2 |
| L9×T2 | 0.24 | 0.31 | -5.71 | 0.03 | 0.57 | 9.28 | 11.28 | 0.13 | -0.03 | -619.1^*^ |
| L10×T2 | -0.63 | -0.82 | -1.63 | -0.31 | -0.01 | 6.36 | 7.53 | -0.03 | 0.01 | -282 |
| L11×T2 | 0.20 | 0.14 | -1.04 | 0.32 | -0.09 | 12.95^*^ | 9.12 | -0.18 | 0.03 | 258.3 |
| L12×T2 | -1.38^*^ | -1.11^*^ | 0.33 | 0.11 | 0.24 | 1.49 | -3.30 | -0.16 | -0.04 | 358.6 |
| L13×T2 | -0.01 | 0.18 | 1.83 | 0.15 | 0.07 | -2.59 | -8.13 | -0.05 | -0.01 | -76.8 |
| L14×T2 | 0.28 | -0.03 | 3.66 | 0.24 | 0.16 | -7.84 | -14.8 | 0.07 | -0.04 | -104.4 |
| L15×T2 | -0.38 | -0.19 | 1.66 | -0.43 | -0.43 | -3.18 | -10.13 | -0.26^*^ | 0.02 | **557.4^*^** |

continued

| Crosses | DYSK | DYAN | PLHT | RAT1 | RAT2 | CO1 | CO2 | EASP | EPP | YLD |
| --- | --- | --- | --- | --- | --- | --- | --- | --- | --- | --- |
| L16×T2 | 0.03 | -0.15 | 1.66 | -0.10 | -0.05 | 6.03 | 21.82^*^ | -0.16 | -0.03 | 102.7 |
| L17×T2 | -0.47 | -0.53 | 0.91 | 0.82^***^ | 0.49 | 6.70 | 7.62 | -0.01 | -0.07 | -171.6 |
| L18×T2 | -0.01 | 0.10 | -4.25 | -0.01 | 0.24 | 8.32 | 10.45 | -0.10 | -0.05 | -185.7 |
| L19×T2 | 0.49 | 0.14 | -2.00 | 0.4 | 0.87^*^ | 2.82 | 23.16^*^ | 0.05 | -0.05 | -166.7 |
| L20×T2 | 0.70 | 0.77 | 2.96 | -0.43 | -0.80^*^ | -11.59 | -4.88 | -0.08 | 0.04 | **600.9^*^** |
| L21×T2 | 1.03^*^ | 1.10^*^ | -0.96 | 0.36 | 0.57 | -2.18 | -9.63 | -0.08 | -0.06 | -63.8 |
| L22×T2 | -0.01 | -0.23 | 0.12 | -0.22 | -0.34 | 1.20 | 3.74 | -0.01 | -0.02 | 113.4 |
| L23×T2 | 0.08 | 0.14 | 4.33 | -0.06 | 0.28 | -4.39 | 4.37 | -0.08 | -0.04 | 419.3 |
| L24×T2 | -0.38 | -0.28 | 0.41 | 0.32 | -0.47 | -5.26 | -12.68 | -0.05 | 0.05 | 511 |
| L25×T2 | -0.76 | -0.53 | 10.04 | -0.18 | -0.72^*^ | -6.76 | -11.97 | -0.16 | -0.06 | **575.4^*^** |
| L26×T2 | -0.17 | -0.19 | 0.21 | -0.06 | -0.30 | 0.20 | 2.74 | 0.15 | -0.06 | -345.4 |
| L27×T2 | -0.80 | -0.48 | -2.04 | -0.22 | -0.13 | -4.22 | -6.01 | 0.11 | 0.04 | -132.4 |
| L28×T2 | 1.74^***^ | 1.56^**^ | -8.21^*^ | -0.31 | -0.34 | -7.18 | -12.13 | 0.34^**^ | -0.03 | -506.4 |
| L29×T2 | 0.08 | 0.52 | -10.2^**^ | 0.07 | -0.26 | -1.09 | 1.91 | 0.26^*^ | 0.49^†^ | -968.1^***^ |
| L30×T2 | 0.70 | 0.48 | -8.21^*^ | 0.11 | 0.7 | 10.24 | 5.03 | 0.15 | 0.00 | -618.0^*^ |
| L1×T3 | -0.20 | -0.41 | 2.56 | 0.24 | 0.42 | 4.76 | 1.29 | 0.00 | 0.03 | 101.3 |
| L2×T3 | 0.21 | 0.13 | 0.47 | 0.12 | 0.46 | -2.90 | -1.00 | -0.23 | 0.04 | 400.6 |
| L3×T3 | -0.20 | -0.37 | 0.76 | -0.42 | -0.58 | -6.11 | -4.71 | -0.23 | 0.03 | 533.8 |
| L4×T3 | -0.12 | -0.08 | 1.89 | -0.13 | -0.12 | 1.81 | -1.38 | 0.07 | -0.00 | 23 |
| L5×T3 | 0.17 | -0.20 | -3.82 | -0.21 | -0.46 | 0.89 | 6.04 | 0.02 | 0.00 | 31.8 |
| L6×T3 | 0.21 | 0.13 | -9.69^*^ | 0.08 | 0.50 | -3.07 | 1.62 | 0.03 | -0.02 | -524.7 |
| L7×T3 | -0.41 | -0.24 | -5.57 | 0.54^*^ | 0.25 | 1.76 | 13.75 | 0.04 | 0.01 | -295.1 |
| L8×T3 | -0.52 | 1.13 | -6.69 | 0.33 | 0.38 | 17.22^**^ | 11.12 | 0.38^**^ | -0.14 | -634.0^*^ |
| L9×T3 | -0.04 | -0.16 | 5.43 | -0.05 | -0.04 | -7.40 | -9.33 | 0.02 | 0.02 | 140.6 |
| L10×T3 | 0.46 | 0.47 | -3.86 | 0.12 | -0.12 | 3.06 | -2.08 | 0.17 | 0.04 | -66.9 |
| L11×T3 | -0.20 | -0.33 | 0.47 | 0.12 | 0.54 | -5.24 | -2.00 | 0.21 | -0.03 | -194.5 |
| L12×T3 | 0.46 | 0.43 | 2.47 | -0.09 | 0.25 | 1.93 | 8.46 | 0.04 | 0.04 | -136.8 |
| L13×T3 | -0.66 | -0.78 | 2.10 | -0.05 | -0.29 | -1.78 | -4.25 | -0.10 | 0.02 | 266.8 |
| L14×T3 | -0.12 | 0.13 | -2.07 | -0.09 | -0.46 | -4.78 | -11.4 | -0.10 | 0.06 | 84.2 |
| L15×T3 | 0.21 | -0.03 | 4.81 | 0.37 | 0.59 | 0.39 | 11.62 | 0.00 | -0.06 | -248.5 |
| L16×T3 | -0.62 | -0.62 | 0.81 | -0.30 | -0.41 | -7.03 | -18.4 | -0.14 | 0.01 | 401.6 |
| L17×T3 | -0.12 | -0.12 | 4.43 | -0.38 | -0.50 | -2.49 | -6.00 | 0.19 | 0.05 | 6.3 |
| L18×T3 | -0.16 | -0.12 | 1.39 | 0.04 | -0.12 | -5.86 | 5.83 | -0.08 | 0.02 | 139.8 |
| L19×T3 | 0.34 | 0.30 | 3.14 | -0.42 | -0.75^*^ | -13.4^*^ | -32.30^**^ | 0.00 | 0.01 | 46.2 |
| L20×T3 | -0.45 | -0.45 | 3.10 | -0.38 | -0.29 | -9.28 | -15.50 | 0.00 | -0.01 | 120.9 |
| L21×T3 | 0.01 | -0.12 | -3.44 | -0.21 | -0.41 | -2.49 | 3.30 | 0.13 | 0.05 | -88.8 |
| L22×T3 | -0.41 | -0.08 | 3.89 | 0.20 | 0.42 | 4.14 | 10.40 | 0.00 | 0.06 | 390.6 |
| L23×T3 | -0.58 | -0.58 | -5.28 | 0.12 | -0.08 | 18.06^**^ | 14.80 | -0.12 | 0.01 | -34.9 |
| L24×T3 | 1.21^*^ | 1.13^*^ | -1.57 | -0.13 | 0.42 | 4.18 | 6.10 | -0.04 | -0.10 | -101.4 |
| L25×T3 | 0.59 | 0.51 | -7.57 | -0.38 | -0.08 | 0.43 | 4.20 | -0.02 | 0.14 | -11 |
| L26×T3 | -0.20 | 0.09 | 1.35 | -0.01 | -0.04 | 8.76 | 1.40 | -0.02 | 0.05 | -17.7 |
| L27×T3 | 0.55 | 0.30 | -0.78 | 0.33 | 0.38 | -1.53 | -0.80 | -0.06 | -0.07 | -59.7 |

Continued

| Crosses | DYSK | DYAN | PLHT | RAT1 | RAT2 | CO1 | CO2 | EASP | EPP | YLD |
| --- | --- | --- | --- | --- | --- | --- | --- | --- | --- | --- |
| L28×T3 | -0.54 | -0.66 | 4.31 | -0.01 | 0.17 | 8.64 | 16.00 | -0.27 | -0.03 | 18.3 |
| L29×T3 | 0.17 | 0.05 | 4.93 | 0.37 | 0.25 | 1.60 | -9.10 | 0.07 | -0.30^***^ | -117.5 |
| L30×T3 | 0.55 | 0.51 | 2.06 | 0.29 | -0.29 | -4.32 | 2.50 | -0.02 | -0.00 | -174.6 |
| SCAse | 0.50 | 0.47 | 4.17 | 0.24 | 0.37 | 6.54 | 10.80 | 0.12 | 0.09 | 286.4 |
| se(S_ij_-S_ki_) | 1.83 | 1.67 | 14.80 | 0.90 | 1.10 | 23.10 | 35.20 | 0.50 | 0.30 | 1053.0 |

*, **, ***, † Significant at p < 0.05, 0.01, 0.001 and 0.0001 levels, respectively. DYSK= Days to 50% silking; DYAN= Days to 50% anthesis; PLHT= Plant height (cm); RAT1 and RAT2= *Striga* damage rating (on a scale of 1-9) at 8 and 10 WAP, respectively; CO1 and CO2= *Striga* emergence count at 8 and 10 WAP, respectively; EASP = ear aspect (rating at a scale of 1-5); EPP = ears per plant and YLD = grain yield (kg/ha).
