# Supplementary figures and images for: Identifying suitable tester for evaluating Striga resistant lines using DArTseq markers and agronomic traits
Source: PLoS One. 2021 Jun 18;16(6):e0253481. doi: 10.1371/journal.pone.0253481 (PMC8213128; doi:10.1371/journal.pone.0253481)

S1Fig.


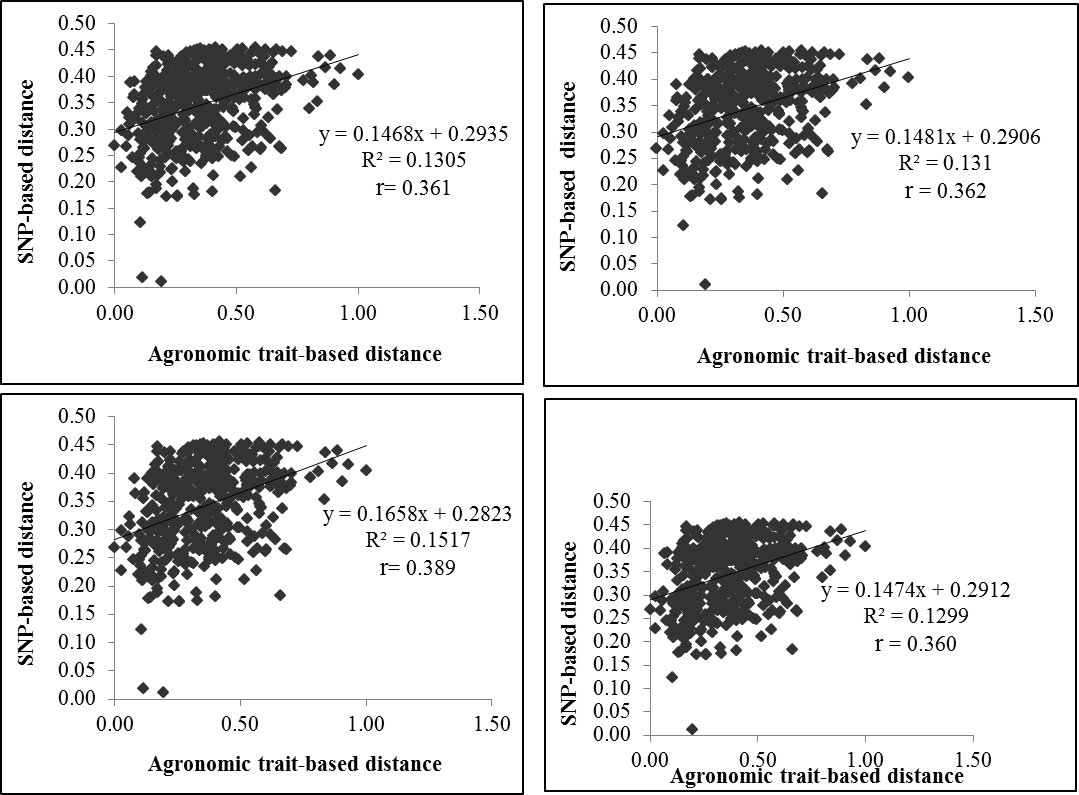


D)

C)

B)

A)

Supplement: S1 Fig — (DOCX) [file pone.0253481.s005.docx]
