# Supplementary material for: Identifying suitable tester for evaluating Striga resistant lines using DArTseq markers and agronomic traits
Source: PLoS One. 2021 Jun 18;16(6):e0253481. doi: 10.1371/journal.pone.0253481 (PMC8213128; doi:10.1371/journal.pone.0253481)
Supplement: Attachment — Submitted filename: Response to Reviewers.docx [file pone.0253481.s006.docx]

**Response to Reviewers**

**Reviewer #1**: This study is useful and novel for evaluating the Striga Resistant Lines using suitable tester. Authors used DArTseq
Markers and agronomic traits to identify the suitable tester. The stud is well supported with both sequences based and field-based study clearly demarcating the suitable tester for evaluating the Striga Resistant Lines. I recommend this article to publish in your journal. However, I have found few spelling mistakes in the article are as following.

**Question**
There should be a space after genus name S. hermonthica Line 69 and Line 225, Line 291
Tester spelling misspelled in line 243
Furthermore spelling is misspelled in line 465

**Response**: All issues concerning spelling errors and space between phrases and words has been checked.

**Reviewer #2: Comments:**

1. Please mention details of T1 in the Abstract.

**Response**: The details of T1 has been introduced as tolerant tester
2. In Table-3 as you have compared agronomic traits, add morphological
pictorial parameter to support your data.

**Response**: Some morphological parameters are included in the result section**.**

3. Reframe the conclusion section for better understanding of the reader.

**Response**: Some modifications has been done in the conclusion section

4. You should perform in vitro or in vivo studies to see If any adverse effects of these resistant strains for future commercial use.
**Response**: Studies showed there is a positive association between in vivo and in vitro studies on resistant strains the parasite for *Striga* attachment count. E.g., Assessment of reactions of diverse maize inbred lines to *Striga* *hermonthica* (Del.) Benth (Menkir 2006)
5. In line 434-435, you should include relevant data.

**Response**: List of crosses with high SCA in the two testers have been included.

6. You need to use some more markers along with SNP marker?

**Response**: It is beyond this study

**Reviewer #3:**

The manuscript entitled “Identifying Suitable Tester for Evaluating Striga Resistant Lines using DArTseq Markers and Agronomic Traits”, has been extensively reviewed by me and found that Authors have developed Tester which has resistance to Striga. Manuscript is good but there are major concerns in the study which needs to be addressed. There are more typological errors in the whole document and needs to be improved in the revision. Manuscript needs to be improved rigorously and based on my analysis I recommend major revision. I consider this manuscript only when all the queries will be answered thoroughly.

Minor Comments:
1. Maize (Zea mays L; 2n = 2x = 20) is one of the most important cereal crops worldwide by 60 providing food, feed and bioenergy [1, 2]…..correct the sentences.

**Response**: the sentence under this statement has been improved
2. 69 affected areas [9]. Inbred lines that combine high yield with resistance to S.hermonthica can be…..there should be space.

**Response**: space has been provided between S. and hermonthica
3. 84 Smith [21] and Hallauer et al. [13] concluded that a homozygous recessive inbred line with a low 85 frequency of desirable dominant genes and favourable alleles would be the most effective tester to 86 identify new inbred lines with high frequency of favourable alleles in testcrosses……lines are not clear improve this.

**Response**: the sentence under this statement has been improved

4. 88 [22, 13]. Mwimali et al. [23] found two Busseola fusca resistant single-crosses as desirable testers…scientific name should be italics.

Response: the scientific name of Busseola fusca has been Italicized in L88.
5. 121 The present studies were, therefore, conducted to characterize Striga resistan…..it should be……The present study is….

**Response**: the statement under L121 has been corrected
6. 138 for DNA extraction. Leaves were collected from 4 to 15 leaves of each line and tester and then 139 bulked. The samples…..my question is that why there is need to bulk the DNA.

**Response**: The DNA was not bulked rather the leaf samples collected from each inbred lines and testers were bulked. To avoid confusion, we rephrased the statement.
7. 179 first standardized in SAS version 9.4 [47]. Correlation among the different traits were analyzed… It should be traits was…

**Response**: the statement under L185 has been corrected
8. The principal component analysis was computed in SAS using the correlation
181 matrix of trait means-centered averaged over environments….write the full form of SAS software.

**Response**: SAS is written in full form in L187.
9. 225 level of reactions to S.hermonthica ranged from 0.02 to 0.45 with an average of 0.36….scientific name should be with space.

**Response**: Space has been provided between S.hermonthica as *S. hermonthica*
10. 295 The performances of the three tester for the traits measured across environments are presented in 296 Table 3…language written is very simple inprove the sentence.

**Response**: The sentence in this line has improved by adding some technical words and phrases.
11. 164 Population structure analysis was performed from 6081 SNPs and a physical distance of 11 kb. 165 between adjacent markers…sentences is incomplete.

**Response**: The sentence has completed by rephrasing the whole statement.

**Major Comments:**
12. Why authors have not used SSRs markers as they are also widely distributed in the genome? **Response**: This was due to a large increase in throughput, independence of DNA sequence information and low cost. The cost advantage compared to marker-by-marker methods is also dramatic.

13. 133 tolerant and susceptible reactions to the parasite, under Striga infested and Striga noninfested 134 conditions at Abuja and Mokwa in Nigeria for two years. …Authors have used only two …I always see it should be 3 years….I thinks there will be biased results, chances of association of false SNPs are there….explain it?

**Response**: We conducted this research in two locations and two years giving a total of four environments, so the chance of getting biased results which will lead to association of false SNPs would be low.
14. The authors have used only 30 samples ….which I think it’s the only major drawback of the manuscript….at least 96 is required to find the marker trait association….. add more samples,,,,because with 30 samples you will get false results.

**Response**: The main objective of this study was to identify suitable tester/s to evaluate Striga resistant inbred lines. Though the sample numbers were limited, we used DArTseq SNP markers to see the usefulness of these markers in achieving the main objective in addition to determining the extent of genetic diversity.

15. The samples were sent to 145 the Diversity Array technology company [38]. All protocols including Library construction, 146 sequencing, and SNP calling were performed at the Diversity Arrays facility, Canberra, Australia…..write all the protocols in the methods…

**Response**: The protocols performed in DArT for library construction, SNP calling and sequencing are briefly explained from L147-152.
16. 192 genotypes. Gene diversity from 6081 SNP markers used to genotype 30 inbred lines and three 193 testers ranged from 0.10 to 0.50 in which 34% of the loci had values between 0.095-0.295 while 194 66 % of them fell in the range of 0.297-0.500 with an average of 0.35. These results indicate high 195 genetic divergence of the Striga resistant inbred lines and testers..Gene diversity value is very low….which again due to less number of samples.

**Response**: The sample numbers were small but the association of marker information and agronomic trait information provided us good result for future breeding programmes and also the main objective of this study was to identify effective tester/s to evaluate *Striga* resistant inbred lines in addition to studying the extent of genetic diversity.
17. The level of heterozygosity found in about 92% of the inbred lines
200 varied from 0.00 to 0.25 with only 8% of the lines having heterozygosity values ranging from 0.25 201 to 1.00. Further analyses revealed that nearly 33% of the markers had observed heterozygosity 202 (Ho) values of 0.00 to 0.05 with only one locus having a heterozygosity value of 1.00, signifying 203 that most of the loci were fixed. The He value also ranged from 0.012 to 0.50 with a mean of 0.35. 204 The fixation index (F) varied significantly from -1.00 to 1.00 with a mean of 0.66, suggesting that205 the presence of higher levels of homozygosity within the lines that were studied….value of heterozygosity is also very low…. which showed your samples are rich in homozygosity.

**Response**: Yes, the inbred lines are advanced lines generated from several generations of selfing.
18. Legend to figures come in the end,,,,improve it.

**Response**: The description of the legends for Fig 1, 2 and 5 has been improved.
19. There are lots of typological errors in the whole manuscript, correct them?

**Response**: All typological errors are carefully checked and grammatical corrections were done by colleague from English speaking country.
20. Discussion and conclusion needs to be strengthened?

**Response**: The discussion and conclusion section of the manuscript has be strengthened by adding more information and explanations.
21. In reference section scientific names needs to be italics, authors have to be serious while submit revise manuscript?

**Response**: All scientific name inside the main body and reference section has been italicized

**Additional point**

We note that you have included the phrase “data not shown” in your manuscript. Unfortunately, this does not meet our data sharing requirements

For this phrase, we have provided supplementary table in the manuscript and the phrase data not show is removed.
