# Supplementary material for: Identifying suitable tester for evaluating Striga resistant lines using DArTseq markers and agronomic traits
Source: PLoS One. 2021 Jun 18;16(6):e0253481. doi: 10.1371/journal.pone.0253481 (PMC8213128; doi:10.1371/journal.pone.0253481)
Supplement: Attachment — Submitted filename: Response to reviewers 2.docx [file pone.0253481.s007.docx]

**Dear Editor,**

We have carefully revised the first version of our manuscript and made some changes based on the reviewer’s recommendation. The comments were very useful to revise our manuscript.

We are glad to send our manuscript titled “Identifying Suitable Tester for Evaluating *Striga* Resistant Lines using DArTseq Markers and Agronomic Traits**”** for publication in **PLOS ONE**.

Here are the responses

**Editor feedback**
Line 28-30 revise the sentence to make it clearer it is confusing or beak it into two.

Response: The sentence broken into two and articulated in a clear manner.

Line 31: "marker-based distance estimates (r= 0.389, P= 0.01) was observed for when T2 has paired" remove grammatical errors.

Response: The grammatical errors in line 31 have been removed by removing unnecessary words and adding appropriate words and prepositions.

Key Words: "mantel test" should be " Mantel test".

Response: it has been changed according to the editor comment.

Lines 62-65: which 81 lines please clear, also revise sentence.

Response: The statement under the indicated lines has been revised by removing inappropriate words, articles and phrase.

Lines 116-118:  "generate 90 test crosses, which were evaluated along with two checks" are these 90 samples were evaluated???

Response: Yes, these 90 samples are testcrosses obtained from crossing of 30 Striga resistant inbred lines and three testers with varying levels of resistance to *Striga hermonthica* for assessing the agronomic performance. A phrase has been added to make the information clear and why this is done in line 118.

line 163: "from the 30 yellow Striga maize inbred lines and three testers" only 30 maize inbred lines and three testers were evaluated?????

Response: The 30 yellow Striga maize inbred lines and three testers were genotyped for assessing genetic diversity

both these statements in lines 116-118 and line 163 are conflicting please check where is the error and make a correlation in study.

Response: Correction has made on line 118 to avoid confusion.

 You are using the term "DArT-seq" however, standard term is "DArT" check care and use appropriate term.

Response: Regarding DArT-seq, we used DArTseq (NGS based genotyping) is a newer version of the older DArT (hybridization based) platform. So we have changed the form accordingly.

Language requires careful revision throughout manuscript, therefore, revise the manuscript thoroughly.

Response: We have revised the grammatical errors as much as we can throughout the manuscript.

Reviewer’s Comments

Reviewer #1: 
Line 21 Striga should be in italics.

Response: The word Striga is in italic form

Line 52 & Line 210 S.hermonthica there should be space after genus name

Response: A space has been provided between S.hermonthica throughout the manuscript as *S. hermonthica*

Line 71 Busseola fusca the name of species should be in italics

Response: The species name of Busseola fusca is italized in line 71
Line 123 -80°c here symbol of celcius in capital (°C)

Response: The symbol of degree Celsius in line 121, C is capitalized and shown in the track change

Reviewer #2

The author have addressed all the raised issues and incorporated changes wherever suggested in previous review. So article can be accepted for publication, however, it is advised to proofread article to remove linguistic errors. Further advised to add more markers along with SNP markers for future studies.

Response: though there is no much comments for reviewer 2, we will see the comparative advantage of SNP markers in our future study

Reviewer #3:

minor mistakes and typological errors which can be corrected at the time of proofreading.

Response: Most typological errors have been addressed as much as possible in this revised version.
